# Persistent mental and physical health impact of exposure to the September 11, 2001 World Trade Center terrorist attacks

**DOI:** 10.1186/s12940-019-0449-7

**Published:** 2019-02-12

**Authors:** Hannah T. Jordan, Sukhminder Osahan, Jiehui Li, Cheryl R. Stein, Stephen M. Friedman, Robert M. Brackbill, James E. Cone, Charon Gwynn, Ho Ki Mok, Mark R. Farfel

**Affiliations:** 1 World Trade Center Health Registry, New York City Department of Health and Mental Hygiene, New York, NY USA; 2 Present Address: Bureau of Tuberculosis Control, New York City Department of Health and Mental Hygiene, 42-09 28th St., CN-72B, Long Island City, Queens, NY 11101 USA; 3 Department of Child and Adolescent Psychiatry, Hassenfeld Children’s Hospital at NYU Langone, One Park Avenue, room 7-314, New York, NY USA; 4 Division of Epidemiology, New York City Department of Health and Mental Hygiene, Long Island City, NY USA

**Keywords:** September 11 terrorist attacks, Epidemiology, Quality of life, Asthma, Depression, Stress disorders, post-traumatic, Gastroesophageal reflux, Registries, Health surveys, Health services

## Abstract

**Background:**

Asthma, gastroesophageal reflux disease (GERD), posttraumatic stress disorder (PTSD) and depression have each been linked to exposure to the September 11, 2001 World Trade Center (WTC) terrorist attacks (9/11). We described the prevalence and patterns of these conditions and associated health-related quality of life (HRQOL) fifteen years after the attacks.

**Methods:**

We studied 36,897 participants in the WTC Health Registry, a cohort of exposed rescue/recovery workers and community members, who completed baseline (2003–2004) and follow-up (2015–16) questionnaires. Lower respiratory symptoms (LRS; cough, dyspnea, or wheeze), gastroesophageal reflux symptoms (GERS) and self-reported clinician-diagnosed asthma and GERD history were obtained from surveys. PTSD was defined as a score > 44 on the PTSD checklist, and depression as a score > 10 on the Patient Health Questionnaire (PHQ). Poor HRQOL was defined as reporting limited usual daily activities for > 14 days during the month preceding the survey.

**Results:**

In 2015–16, 47.8% of participants had ≥1 of the conditions studied. Among participants without pre-existing asthma, 15.4% reported asthma diagnosed after 9/11; of these, 76.5% had LRS at follow up. Among those without pre-9/11 GERD, 22.3% reported being diagnosed with GERD after 9/11; 72.2% had GERS at follow-up. The prevalence of PTSD was 14.2%, and of depression was 15.3%. HRQOL declined as the number of comorbidities increased, and was particularly low among participants with mental health conditions. Over one quarter of participants with PTSD or depression reported unmet need for mental health care in the preceding year.

**Conclusions:**

Nearly half of participants reported having developed at least one of the physical or mental health conditions studied by 2015–2016; comorbidity among conditions was common. Poor HRQOL and unmet need for health were frequently reported, particularly among those with post-9/11 PTSD or depression. Comprehensive physical and mental health care are essential for survivors of complex environmental disasters, and continued efforts to connect 9/11-exposed persons to needed resources are critical.

## Introduction

On September 11, 2001, terrorists launched two hijacked commercial jet planes into the World Trade Center (WTC) complex in Manhattan. In addition to immediately claiming over 2700 lives [1], the destruction of the WTC towers and nearby structures exposed hundreds of thousands of survivors to massive quantities of dust and fumes from collapsing buildings, the combustion of jet fuel, and lingering fires [2, 3], and to extreme psychological trauma [4]. Exposures to environmental hazards and psychological stressors continued during the months of rescue and recovery work that followed.

These experiences have had an enduring effect on the health of many 9/11 survivors. Respiratory symptoms were first described among heavily exposed firefighters [5], and subsequently documented among other rescue/recovery workers and lower Manhattan area community members [6–10]. Several other chronic aerodigestive disorders, including gastroesophageal reflux disease (GERD), have since been linked to 9/11-related exposures [10–12]. Respiratory conditions and GERD have persisted for many of those affected, necessitating long-term follow-up and, often, chronic medication use.

The events of 9/11 have also taken a significant toll on the psychological health of many who were exposed [4, 6]. Posttraumatic stress disorder (PTSD), which was found to be common among survivors during the first few years following 9/11, has persisted for many years for a substantial portion of survivors [13, 14]. Depression, often accompanying PTSD, is also common in this population [15, 16].

Considerable overlap across multiple 9/11-related conditions complicates the medical treatment and course of these conditions [17–21]. Having more than one of these conditions is associated with poorer outcomes [17, 18]. The combined effect of multiple, concurrent 9/11-related conditions was associated with low quality of life and productivity more than a decade after 9/11 [22, 23].

Updated information on the prevalence and patterns of the most common 9/11-related conditions and on the relationship between these conditions and quality of life is needed to inform the provision of health care for persons who were directly exposed to 9/11. We assessed the prevalence of asthma, GERD, PTSD and depression in 2015–16 among members of the World Trade Center Health Registry (Registry), a cohort of 9/11-exposed workers and lower Manhattan area community members, and estimated the impact on the total population of directly exposed persons.

## Methods

### Data source and study sample

We used data from the Registry, a voluntary cohort of rescue/recovery workers and volunteers, lower Manhattan area community members, and passersby on 9/11 who were directly exposed to the 9/11 terrorist attacks or subsequent rescue and recovery efforts [6, 24]. The Registry invited potential participants identified from lower Manhattan area building and employer lists (list-identified applicants) or through a broad-based, multi-lingual media campaign (self-identified applicants) to be screened for eligibility. Between September 5, 2003 and November 20, 2004, a total of 71,431 persons who met the eligibility criteria completed an enrollment questionnaire (Wave 1; 95% telephone administered, 5% face-to-face) regarding sociodemographic information, 9/11-related exposures and experiences, and health status and history. Enrollees are invited to provide updated health and quality of life information through periodic surveys, the most recent of which, Wave 4, was conducted in 2015–2016 [25] (questionnaire available at https://www1.nyc.gov/site/911health/researchers/health-data-tools.page). The US Centers for Disease Control and Prevention and New York City Department of Health and Mental Hygiene institutional review boards approved the Wave 1 and Wave 4 protocols.

The current study included enrollees who completed both the Wave 1 enrollment and Wave 4 questionnaires, were ≥ 18 years of age on 9/11/2001; and remained active participants in the Registry as of November 15, 2016.

### 9/11-related exposures

We used self-reported data from the Wave 1 questionnaire to define 9/11-related exposures. We considered several exposures that have been associated with 9/11-related health conditions in previous studies. Participants who reported being directly exposed to the massive cloud of dust and debris that resulted from the collapse of the World Trade Center towers and nearby infrastructure on 9/11 were considered to have dust cloud exposure. 9/11-related injury was defined as the report of incurring a cut, abrasion, or puncture wound; sprain or strain; burn; broken or dislocated bone; or concussion or head injury due to the 9/11 attacks. Personally witnessing a traumatic event was defined as having seen ≥1 of the following: an airplane strike a WTC tower; a building collapse; people running from the area or falling from the towers; or someone being injured or killed.

Enrollees who performed any rescue/recovery work, including volunteers, were considered rescue/recovery workers. Other enrollees were hierarchically categorized as lower Manhattan area residents, area workers (including staff or adult students from area schools), or passersby; for certain analyses, this group was considered collectively as community enrollees. For rescue/recovery workers, duration of work, date of first arrival for work, and whether a participant worked on the WTC dust and debris pile were considered. The latter two factors were operationalized as a single variable: arrived on 9/11 and worked on pile, arrived 9/11 but worked elsewhere, arrived 9/12–17, or arrived 9/18 or later.

### Health and quality of life outcomes

Asthma was defined as clinician-diagnosed asthma reported on the Wave 1 or Wave 4 questionnaire, and GERD as clinician-diagnosed GERD reported on the Wave 4 questionnaire (GERD was not queried at Wave 1). Probable PTSD at Wave 4, subsequently referred to as PTSD for simplicity, was defined as a score of ≥44 on the 17-item PTSD Checklist (PCL-17), which inquired specifically about symptoms that were related to 9/11. Depression at Wave 4 was defined as a score ≥ 10 on the 8-item Patient Health Questionnaire. Lower respiratory symptoms (LRS) were defined as wheezing, dyspnea, or cough reported on > 8 of the 30 days preceding the interview, or use of physician-prescribed inhaler for breathing problems during the preceding 30 days. Gastroesophageal reflux symptoms (GERS) at Wave 4 were defined as heartburn or acid reflux reported at least weekly during the preceding year.

Health-related quality of life (HRQOL) was assessed using the number of days of poor physical or mental health reported during the 30 days preceding Wave 4, and the number of days on which poor health limited a respondent’s usual activities during the same period (each dichotomized at ≥14 days). General health, satisfaction with life, and health care access and utilization were also assessed at Wave 4. Participants were asked whether they had needed mental health care or counseling during the preceding year; if so, they were further asked whether they had received the care or counseling they needed. Those who reported needing care, but not having received it, were considered to have unmet need for mental health care. Similar questions were asked for physical health care, and unmet need was likewise considered reporting having needed, but not received, medical care during the preceding year.

### Statistical analysis

We assessed whether Wave 4 participants differed from those who enrolled at Wave 1 but did not complete Wave 4 in terms of baseline socio-demographic characteristics, 9/11-related exposures, or self-reported health status using chi-square tests. We calculated the lifetime prevalence of asthma and GERD in the complete study sample; the prevalence of post-9/11-diagnosed asthma and GERD among participants without a history of the relevant condition before 9/11; and the prevalence of disease-related symptoms at Wave 4 among participants with post-9/11-diagnosed asthma and GERD. We computed the prevalence of PTSD and depression at Wave 4 among participants without a pre-9/11 diagnosis of the respective condition. We used population estimates from the 2010 US Census (www.census.gov/prod/cen2010/briefs/c2010br-03.pdf) to calculate the age-adjusted prevalence of asthma, GERD, PTSD, and depression at Wave 4. For participants who reported a year of diagnosis on the Wave 4 questionnaire, we calculated the annualized rate of post-9/11 asthma and GERD diagnoses between 2002 and 2015. We calculated the prevalence of indicators of HRQOL, general health and satisfaction, and health care access and utilization among participants with each health condition, and according to the number of health conditions diagnosed or present at the time of Wave 4.

To estimate the prevalence of asthma, GERD, PTSD, and depression among the approximately 409,000 persons who are thought to have been eligible for Registry enrollment [26], we applied the prevalence of post-9/11-diagnosed asthma and GERD and the prevalence of PTSD and depression at Wave 4 to the estimated number of people exposed. We calculated lower bound, mid value and upper bound estimates separately for rescue/recovery workers and community enrollees, using previously-described methods [24, 26]. The lower bound was calculated assuming that, among list-identified enrollees, those with symptoms potentially related to 9/11 exposure enrolled at a rate 50% higher than those who were asymptomatic. Estimates were rounded to the nearest integer.

Analyses were conducted in SAS version 9.4 (SAS Institute, Inc., Cary, North Carolina). For all analyses, 2-sided *P* values were considered significant when less than 0.05.

## Results

Of 67,504 Registry enrollees invited to complete the Wave 4 questionnaire, 36,864 (55%) participated (49% on paper, 51% via Web). Compared to non-participants, a higher proportion of participants were white; were aged 45 or older on 9/11; had completed college or higher education; had an annual family income of $75,000 or above; were co-habiting at Registry enrollment; were self-identified as potentially eligible for the Registry; or had performed rescue/recovery work after 9/11 (Appendix). The proportion exposed to the dust cloud on 9/11 was similar in participants and non-participants (52.1% vs. 51.2%, *P* = 0.02). The proportion of participants and non-participants who had personally witnessed trauma on 9/11 was also very similar (69.5% vs. 69.7%, *P* = 0.63). There was no statistical difference between the prevalence of asthma or GERD in the two groups at Registry enrollment; however, the proportion with PTSD at enrollment was lower in Wave 4 participants (15%) than in non-participants (18%, *P* < 0.01).

From the 36,864 Wave 4 participants, we excluded those who withdrew from the Registry (*n* = 21) after completing Wave 4, had their questionnaire completed by a proxy (*n* = 42), or were less than 18 years of age on 9/11 or missing age data (*n* = 904), resulting in a sample of 35,897 for subsequent analyses.

The lifetime prevalence of asthma was 25.4% (Table 1). Among participants without pre-9/11 asthma (*n* = 31,951), 15.4% were diagnosed with asthma after 9/11. The prevalence of post-9/11 asthma was higher among participants who were self-identified than among participants who were identified from building or employer lists (17.1% vs. 10.4%); among rescue/recovery participants (18.1%) than among other groups of enrollees; and among participants who were exposed to the 9/11 dust cloud than among those who were not (17.6% vs. 13.1%). Among rescue/recovery workers, the prevalence of asthma was higher among those with longer compared to shorter duration of work, and higher among those who had initiated rescue/recovery work on or soon after 9/11 than in those who began participating in rescue-recovery work later.

**Table 1:** Prevalence of asthma and gastroesophageal reflux disease (GERD) among adult World Trade Center Health Registry enrollees in 2015–16

		Asthma						GERD					
	Adult W4 participants	Lifetime prevalence ^a^	Participants without pre-9/11 asthma	Newly diagnosed asthma since 9/11^c^		Persistent LRS among those with post-9/11 asthma ^d^		Age adjusted lifetime prevalence ^e^	Participants without pre-9/11 GERD diagnosis	Newly diagnosed GERD since 9/11^f^		Frequent GERS among those with post-9/11 GERD ^g^	
Characteristic	N	% (age adjusted) ^b^	N	N	% (age adjusted) ^b^	N	%	% (age adjusted) ^b^	N	N	% (age adjusted) ^b^	N	%
Overall	35,897	25.4 (24.7)	31,951	4556	15.4 (14.3)	3318	76.5	25.5 (24.2)	34,552	7119	22.3 (20.7)	5068	72.7
Sex													
Male	21,861	22.9	19,885	2674	14.6	2088	81.2	26.5	21,095	4595	23.6	3353	74.4
Female	14,036	29.3	12,066	1882	16.8	1230	69.6	23.9	13,457	2524	20.4	1715	69.7
Age on 9/11													
18–24	1775	26.6	1485	168	11.7	95	58.6	12.1	1750	181	10.8	131	72.4
25–44	18,830	26.1	16,748	2576	16.3	1831	74.8	24.9	18,285	3870	22.5	2822	74.1
45–64	14,412	24.5	12,920	1739	14.9	1338	80.7	28.1	13,687	2945	23.9	2043	71.3
65+	880	20.7	798	73	11.0	54	79.4	23.9	830	123	18.2	72	62.1
Race/ethnicity													
White	25,160	23.4	22,560	2955	14.0	2096	74.6	26.1	24,123	5128	22.7	3639	72.3
Black/African American	3483	27.8	3035	445	16.1	349	81.4	21.1	3387	566	18.6	382	70.0
Hispanic/Latino	4064	34.8	3471	709	22.5	561	82.1	27.8	3947	900	25.4	684	77.8
Asian	1951	25.1	1791	266	17.3	184	72.4	19.5	1903	278	17.1	181	66.8
Other	1239	28.6	1094	181	18.2	128	78.1	26.6	1192	247	23.4	182	75.5
Education (at Wave 1)													
Less than high school	7450	27.8	6704	1132	18.8	933	86.1	30.0	7206	1763	27.3	1313	77.3
At least some college	8821	27.1	7891	1301	17.8	1034	83.1	29.7	8505	2110	26.9	1560	75.3
College or higher	19,347	23.7	17,105	2084	13.0	1319	66.8	22.0	18,572	3199	18.5	2162	68.6
Income (at W1)													
Less than $25,000	2681	32.4	2343	441	21.3	337	82.4	24.2	2609	494	21.8	370	78.7
$25,000 - $74,999	13,408	26.6	11,901	1820	16.6	1346	77.5	25.7	12,908	2673	22.5	1921	73.4
$75,000 - $150,000	12,303	24.5	11,057	1594	15.4	1157	75.6	27.4	11,842	2711	24.4	1937	72.8
$150,000 or more	4048	21.0	3556	314	9.4	196	66.4	20.4	3882	614	16.8	417	68.7
Smoking status (wave 1)													
Never	20,375	25.4	18,107	2565	15.3	1782	73.6	24.1	19,711	3881	21.3	2746	72.1
Former	10,270	25.4	9148	1296	15.4	973	78.2	28.3	9768	2177	24.3	1532	72.2
Current	5022	25.2	4497	659	15.8	537	83.9	25.6	4854	1020	22.8	761	76.2
Self identified													
Yes	26,806	27.2	23,749	3772	17.1	2744	76.4	27.4	25,770	5782	24.3	4163	73.4
No	9091	19.9	8202	784	10.4	574	76.8	19.8	8782	1337	16.7	905	69.6
Registry eligibility group													
Rescue/recovery worker	16,898	26.2	15,327	2579	18.1	2016	81.6	30.9	16,324	4290	28.3	3153	75.1
Lower Manhattan area resident	4847	24.7	4255	511	13.2	327	68.3	19.7	4680	701	16.5	462	67.3
Lower Manhattan area worker/student	12,517	24.1	10,964	1264	12.5	843	70.4	20.7	11,987	1862	16.9	1265	69.5
Passerby	1400	28.5	1202	175	15.8	116	70.3	21.3	1338	214	17.3	152	71.7
Dust cloud exposure													
Yes	18,677	27.8	16,526	2683	17.6	2008	78.1	27.4	17,974	4006	24.3	2917	74.4
No	17,070	22.7	15,298	1860	13.1	1298	74.0	23.5	16,433	3090	20.3	2134	70.6
Rescue/recovery workers and volunteers													
Duration of R/R work													
1–7 days	5800	25.0	5120	680	14.3	482	74.6	25.5	5576	1152	22.3	806	71.7
8–30 days	5155	24.6	4740	781	17.6	614	82.1	31.9	4969	1356	29.1	1007	75.6
31–90 days	2799	28.9	2586	542	22.6	446	85.1	37.2	2730	906	35.5	679	76.4
>90 days	2523	30.1	2336	517	24.0	430	86.2	35.5	2456	756	33.6	582	78.7
Date of arrival for R/R work													
9/11 on pile	2536	33.7	2400	666	29.7	567	88.3	48.8	2467	1100	47.3	865	79.7
9/11, other site	2371	28.9	2120	391	19.8	313	82.6	31.8	2297	626	29.4	457	74.3
9/12–9/17	6671	26.1	6049	1006	17.9	773	80.0	30.7	6468	1696	28.3	1248	75.7
9/18 or later	4702	21.3	4215	454	11.6	317	74.1	21.8	4501	752	18.0	508	68.7

*GERD* gastroesophageal reflux disease

*GERS* gastroesophageal reflux symptoms

*LRS* lower respiratory symptoms

^a^Proportion of adult W4 participants who reported clinician-diagnosed asthma at Wave 1 or 4

^b^Age adjusted to the 2010 United States Census

^c^Asthma diagnosed after 9/11/2001 reported at Wave 1 or 4. Denominator is number of participants without pre-9/11 asthma

^d^Persistent LRS defined as cough, wheeze, or shortness of breath for at least 8 of the 30 days preceding Wave 4 completion, or use of a prescription inhaler

^e^Proportion of adult W4 participants who reported clinician-diagnosed GERD at Wave 4

^f^GERD diagnosed in 2002 or later as reported on Wave 4. Denominator is number of participants without pre-9/11 GERD

^g^Frequent GERS at Wave 4 defined as heartburn or acid reflux reported at least weekly during the year before survey completion

Among participants who were diagnosed with asthma after 9/11, most (76.5%) had persistent lower respiratory symptoms or were taking prescription medications for asthma at the time of Wave 4. The proportion with persistent symptoms was higher among participants with dust cloud exposure than among those without (78.1 vs. 74.0%). Among rescue/recovery workers with post-9/11 asthma, the prevalence of persistent lower respiratory symptoms at Wave 4 increased with longer duration of rescue/recovery work and earlier date of arrival for work.

The lifetime prevalence of GERD was 25.5%. Among participants without a pre-9/11 GERD diagnosis, 22.3% were diagnosed with the condition after 9/11; of these, 72.7% reported frequent GERS at the time of Wave 4. The prevalence of post-9/11 GERD was higher among participants who were self-identified than among those who were list-identified (24.3% vs. 16.7%), and among rescue/recovery workers (28.3%) than among other Registry enrollees. The prevalence was also higher among those with dust cloud exposure than among those without (24.3% vs. 20.3%), and, like asthma, was increasingly prevalent with longer duration of rescue/recovery work and earlier date of arrival for work.

The prevalence of PTSD and depression is shown in Table 2. Among participants without a pre-9/11 diagnosis of PTSD, the prevalence of PTSD at Wave 4 was 14.3%. The prevalence was higher among those who were self-identified than among those identified from building or employer lists (15.4% vs. 11.4%, respectively), and among rescue/recovery workers (15.3%) and passersby (15.7%) than among area residents (11.9%) or workers (13.7%). The prevalence was also higher among those who experienced each type of 9/11-related exposure examined than among those who had not experienced the respective exposure (e.g., 16.3% among those who personally witnessed traumatic events vs. 10.1% among those who did not). Among rescue/recovery workers, the prevalence increased with longer duration of work and earlier arrival at the scene.

**Table 2:** Prevalence of posttraumatic stress disorder (PTSD) and depression among adult World Trade Center Health Registry enrollees in 2015–16

	PTSD			Depression		
	Adult Wave 4 participants without pre-9/11 PTSD diagnosis ^a^	PTSD ^b^ at follow-up (2015–2016)		Adult Wave 4 participants without pre-9/11 depression diagnosis ^d^	Depression ^e^ at follow-up (2015–2016)	
Characteristic	N	N	% (age adjusted) ^c^	N	N	% (age adjusted)^c^
Overall	34,211	4762	14.3 (13.1)	33,111	4709	15.3 (14.5)
Sex						
Male	21,214	2952	14.3	20,713	2959	15.2
Female	12,997	1810	14.4	12,398	1750	15.4
Age on 9/11						
18–24	1689	177	10.8	1645	211	13.5
25–44	17,920	2781	16.0	17,521	2759	16.8
45–64	13,735	1731	13.0	13,115	1653	13.6
65+	867	73	9.1	830	86	12.2
Race/ethnicity						
White	23,917	2813	12.0	22,959	2910	13.5
Black or African American	3351	556	17.2	3328	518	17.2
Hispanic or Latino	3882	889	23.8	3809	790	22.7
Asian	1901	280	15.8	1879	282	16.8
Other	1160	224	20.3	1136	209	20.7
Education (at Wave 1)						
Less than high school	7188	1466	21.3	7091	1380	21.4
At least some college	8434	1402	17.1	8277	1329	17.2
College or higher	18,321	1847	10.4	17,479	1956	11.9
Income (at Wave 1)						
Less than $25,000	2493	614	26.1	2341	518	25.1
$25,000 - $74,999	12,773	2111	17.1	12,361	2065	18.1
$75,000 - $150,000	11,786	1365	11.8	11,483	1440	13.3
$150,000 or more	3845	328	8.7	3716	338	9.6
Smoking status (at Wave 1)						
Never	19,547	2473	13.0	19,058	2408	13.6
Former	9724	1255	13.3	9269	1268	14.7
Current	4724	1002	21.9	4571	1002	23.7
Self identified						
Yes	25,407	3791	15.4	24,655	3633	15.8
No	8804	971	11.4	8456	1076	13.8
Registry eligibility group						
Rescue/recovery worker	16,424	2453	15.3	15,957	2436	16.3
Lower Manhattan area resident	4563	519	11.9	4243	544	14.2
Lower Manhattan area worker/student	11,701	1555	13.7	11,460	1481	13.9
Passerby	1311	200	15.7	1239	209	18.2
Dust cloud exposure						
Yes	17,568	3033	17.8	17,136	2773	17.5
No	16,503	1707	10.6	15,838	1910	12.9
Personally witnessed trauma on 9/11						
Yes	23,132	3643	16.3	22,533	3435	16.4
No	10,527	1034	10.1	10,053	1187	12.6
Rescue/recovery workers and volunteers						
Duration of work						
1–7 days	5589	713	13.1	5355	764	15.2
8–30 days	5035	723	14.7	4884	716	15.5
31–90 days	2732	422	15.9	2704	415	16.4
>90 days	2457	533	22.4	2420	469	21.0
Date of arrival for rescue/recovery work						
9/11 on pile	2471	467	19.3	2470	377	16.2
9/11, other site	2269	406	18.5	2236	387	18.7
9/12–9/17	6496	1081	17.1	6362	1078	18.2
9/18 or later	4581	441	9.9	4304	525	13.0

^a^Self-reported PTSD diagnosed by a clinician in 2001 or earlier, as reported on the 2015–16 Wave 4 questionnaire

^b^Defined as a score > 44 on the 17-item, event-specific PTSD Checklist on the 2015–16 Wave 4 questionnaire

^c^Age adjusted to the 2010 United States Census

^d^Self-reported depression diagnosed by a clinician in 2001 or earlier, as reported on the 2015–16 Wave 4 questionnaire

^e^Defined as a score > 10 on the 8-item Patient Health Questionnaire on the 2015–16 Wave 4 questionnaire

Similar patterns were found for depression. The overall prevalence at Wave 4 was 15.3%, and was higher in those who were self-identified than among those who were not (15.8% vs. 13.8%); in rescue/recovery workers (16.3%) and passersby (18.2%) than among area residents (14.2%) or workers (13.9%); and among those who had been exposed to the 9/11 dust cloud or witnessed traumatizing events than among those who had not (17.5% vs. 12.9, and 16.4% vs. 12.6%, respectively). There was a suggestion of an increase in the prevalence of depression with increasing duration of rescue/recovery work, but no clear pattern in the prevalence of depression according to the date of arrival for work.

We examined patterns of overlap in the prevalence of the four 9/11-related conditions studied among 30,958 participants with complete data on the presence or absence of each condition. Nearly half of these participants (48.8%) had developed at least one of these conditions by Wave 4. The most common patterns were asthma alone (10.9% of participants with complete data); GERD alone (10.8%); asthma and GERD (5.8%); PTSD and depression (4.2%); depression alone (3.0%); all four conditions (2.5%); and PTSD alone (2.0%; data not shown in tables).

It is estimated that approximately 409,000 persons were eligible for Registry participation [26]. Projection of the Wave 4 questionnaire to the complete simulated population of directly exposed persons yielded estimates of approximately 39,938 cases of asthma; 61,678 of GERD; 46,762 of PTSD; and 52,440 of depression occurring since 9/11 (Table 3). We estimated that 162,070 persons who were directly exposed to the 9/11 WTC attacks (approximately 38,000 rescue/recovery workers and 124,000 community members) had developed one or more of these four conditions by 2015–16.

**Table 3:** Projected number of cases of 9/11-related conditions among the ~ 409,000 persons estimated to have been eligible for World Trade Center Health Registry enrollment ^a, b^

		Asthma			GERD			PTSD			Depression			One or more 9/11-associated conditions ^d^		
Participant group	Total Exposed ^c^	Lower bound	Mid-point	Upper bound	Lower bound	Mid-point	Upper bound	Lower bound	Mid-point	Upper bound	Lower bound	Mid-point	Upper bound	Lower bound	Mid-point	Upper bound
Rescue/recovery workers	91,469	7121	10,281	19,275	12,146	17,085	29,659	7683	11,061	15,347	9573	13,645	15,445	29,406	38,001	50,388
Community enrollees	318,023	20,406	29,657	44,117	31,186	44,593	56,270	23,076	33,402	45,125	26,960	38,795	47,340	95,076	124,068	151,471
All	409,492	27,527	39,938	63,391	43,333	61,678	85,929	30,759	46,762	62,862	36,532	52,440	62,784	124,483	162,070	201,859

GERD: gastroesophageal reflux disease

PTSD: posttraumatic stress disorder

^a^Burden estimates are rounded to the nearest integer

^b^Midpoint estimate is the product of the percent ill among list-identified persons and the total population exposed. Lower bound estimate adjusts for the possibility that even among listed persons, those ill were 50% more likely to enroll in the WTCHR. The lower bound estimate is the product of the total population exposed and the ratio of A to B, where (A) is the number of list-identified persons who were ill and (B) is the sum of the number of list-identified persons who were healthy × 1.5 and the number of list-identified persons who were ill. Upper bound estimate is the product of the percent ill among self-identified persons and the total population exposed

^c^Estimates from Murphy J, Brackbill RM, Thalji L, et al. Measuring and maximizing coverage in the World Trade Center Health Registry. Stat Med 2007;26:1688–1701

^d^Conditions included were asthma, GERD, PTSD, and depression

Participants with each of the four conditions studied reported considerably poorer HRQOL during the 30 days preceding completion of the Wave 4 questionnaire compared to participants with none of these conditions (Table 4). This was particularly pronounced among participants with a mental health condition; 46.6% of those with PTSD and 47.1% of those with depression reported that their health had limited their usual activities during 14 or more of the 30 days preceding Wave 4 completion. Similar patterns were found in measures of general health and satisfaction with life; 47.4% of participants with PTSD and 50.8% of those with depression reported being dissatisfied or very dissatisfied with life, while 64.9% of those with PTSD and 66.0% of those with depression reported fair or poor general health. A high proportion of participants with PTSD reported unmet need for physical (7.4%) or mental health care (25.3%) during the past year. Similarly, among participants with depression, 7.1% reported unmet need for mental health care, and 26% reported unmet need for mental health care.

**Table 4:** Quality of life, health care access, and health care utilization according to presence/absence of post-9/11-onset health conditions

		Health-related quality of life in 30 days before questionnaire				General health and satisfaction				Health care access and utilization during year before questionnaire			
Condition ^a^		> 14 days of poor physical or mental health		Poor health limited usual activity for > 14 days		Dissatisfied or very dissatisfied with life		Fair or poor general health		Unmet need for medical care		Unmet need for mental health care	
	N	n	%	n	%	n	%	n	%	n	%	n	%
Asthma	4556	2091	47.1	1250	28.9	1114	24.9	2232	49.8	182	4.1	604	13.6
GERD	7119	2981	42.8	1721	25.3	1601	22.8	3041	43.2	211	3.1	827	11.9
PTSD	4762	3274	70.4	2117	46.6	2220	47.4	3047	64.9	341	7.4	1169	25.3
Depression	4709	3413	73.9	2130	47.1	2352	50.8	3069	66.0	326	7.1	1192	26.0
None	16,150	2057	13.0	700	4.5	990	6.2	1771	11.1	244	1.5	743	4.6

GERD: gastroesophageal reflux disease

PTSD: posttraumatic stress disorder

^a^Asthma was defined as self-reported, clinician-diagnosed asthma reported on the Wave 1 or Wave 4 questionnaires. GERD was defined as self-reported, clinician-diagnosed GERD reported on the Wave 4 questionnaire. PTSD was defined as a score > 44 on the 17-item, event-specific PTSD Checklist on the 2015–16 Wave 4 questionnaire, among enrollees who did not report a pre-9/11 diagnosis of PTSD. Depression was defined as a score > 10 on the 8-item Patient Health Questionnaire on the 2015–16 Wave 4 questionnaire among enrollees who did not report a pre-9/11 diagnosis of depression

**Fig. 1:**
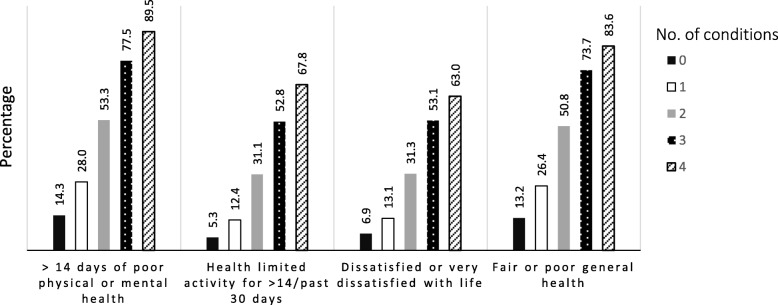
Prevalence of measures of poor health-related quality of life at Wave 4 according to the number of 9/11-related health conditions present. Conditions are asthma, gastroesophageal reflux disease, posttraumatic stress disorder, or depression

**Fig. 2:**
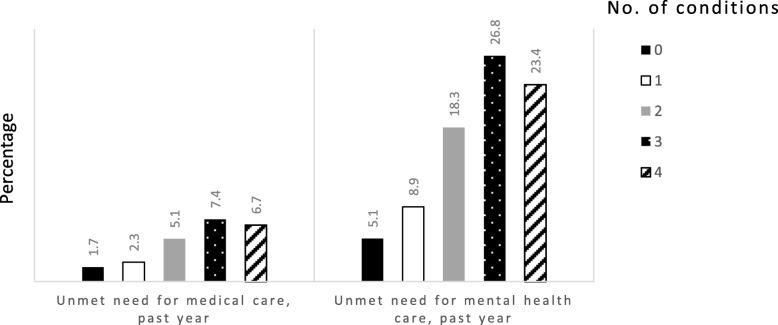
Prevalence of self-reported unmet need for health care at Wave 4 according to the number of 9/11-related conditions present. Conditions are asthma, gastroesophageal reflux disease, posttraumatic stress disorder, or depression

When HRQOL was examined according to the number of health conditions present at the time of Wave 4, the prevalence of each measure of poor HRQOL increased steadily with the number of conditions present (Fig. 1). There was a similar, though less pronounced, pattern when the prevalence of unmet need for health care was studied according to the number of conditions (Fig. 2).

## Discussion

Although more than fifteen years have passed since the September 11, 2001 WTC terrorist attacks, their impact continues to unfold. Nearly half of enrollees in this large cohort of 9/11-exposed rescue/recovery workers and community members reported having developed one or more of the health conditions studied by 2015–16; extrapolation of these findings to the estimated complete population of directly-exposed survivors suggests that more than 162,000 persons are struggling with potentially 9/11-related mental or physical health symptoms today. Comorbidity among conditions was both common and closely tied to poor HRQOL in our study, implying that survivors of complex disasters are likely to require comprehensive, long-term medical follow-up and care. Participants with PTSD or depression reported the worst HRQOL and the highest levels of unmet need for mental health care, suggesting that increased efforts to reach this potentially vulnerable group are needed.

At 24.7%, the age-adjusted lifetime prevalence of asthma in our study was much higher than asthma prevalence estimates among adults reported in the 2015 National Health Interview Survey (12.7%) [27] and the 2014 New York City Community Health Survey (11.3%) [28], which measured self-reported clinician-diagnosed asthma using methods similar to the Registry’s. The age-adjusted point prevalence of PTSD in our sample, 13.1%, was also substantially higher than available population-based estimates, including the on-line 2011 National Stressful Events Survey (5.1% in past 6 months) [29] and the face-to-face 2001–2003 National Comorbidity Survey (6.1%) [30], although each of these measured PTSD differently than we did. Additionally, depression was more common in our sample (age-adjusted prevalence 14.5%) than in studies of the general population that used a similar version of the PHQ to define the condition (8.3%, 2013–14 New York City Health and Nutrition Evaluation Survey [31]; 7.6%, 2009–2012 National Health and Nutrition Evaluation Survey [32]). We did not find population-based estimates for GERD that were measured comparably to ours. Our findings are broadly consistent with illness prevalence reports from other 9/11-exposed cohorts [10, 33, 34].

Each of the health conditions examined in this study was associated with poor HRQOL, but this association was particularly evident for the mental health conditions, with almost two thirds of participants with PTSD or depression reporting poor HRQOL during the month preceding completion of the questionnaire. HRQOL also declined steadily as the number of co-morbid conditions increased, consistent with previous studies [18, 22]. The close relationship between the mental health conditions studied and poor HRQOL may be due, largely, to the fact that PTSD and depression were usually present in combination with other comorbidities [22, 35]. Nonetheless, our results suggest that the toll associated with active PTSD and depression is substantial for society as well as for affected individuals, since both of these conditions were associated with frequent limitation of daily activities, and thus likely with a considerable decrease in productivity and other contributions.

Although health care for 9/11-related conditions is offered at no out-of-pocket cost to 9/11-exposed persons through the World Trade Center Health Program, many participants in this study reported unmet need for health care during the preceding year. The survey inquired about medical and mental health care in general, and did not specifically query whether the unmet need was for a condition that may be 9/11-related, so it is likely that some of the unmet needs reported were for other conditions. Nonetheless, the finding that 25% of patients with PTSD and 26% of those with depression reported a recent unmet need for mental health care is striking when compared to that of the general NYC population (13% [36]). These finding suggests that a more focused approach is needed to identify and address barriers that prevent enrollees with mental health symptoms from obtaining care. A previous study of such barriers in this cohort found that greater severity of mental health symptoms, a lack of health insurance, and lower levels of social support were associated with unmet need for mental health care [37]. Targeted outreach to individuals for whom these barriers are likely to exist, through programs such as the Registry’s Treatment Referral Program (which conducts personalized outreach to refer eligible enrollees to care through the WTC Health Program), is a crucial step toward improving access.

These results must be viewed in light of the fact that all data, including 9/11-related exposures and health outcomes, were self-reported, and that PTSD and depression were defined using screening instruments. Although we used tools such as the PCL-17 and PHQ that have been used extensively, including in multiple previous studies of 9/11-related health outcomes, estimates of the prevalence of conditions presented here are not a substitute for objectively collected clinical data. Instead, our results complement studies that include medically verified diagnoses by reflecting the ongoing needs of a larger and more diverse panel of 9/11 survivors than could be assessed otherwise. An additional consideration is that, while the Registry is the largest and most diverse cohort of 9/11-exposed persons in existence, it is a voluntary study that is estimated to have enrolled a minority of those exposed (17% [26]), and therefore may not be representative of the full spectrum of 9/11-exposed persons.

Approximately 55% of eligible Registry enrollees participated in the Wave 4 questionnaire, with enrollees who reported being white, highly educated, having a higher family income, or performing 9/11 rescue/recovery work more likely to participate in the Wave 4 suvey than their counterparts. Additionally, those who initially self-identified for screening for Registry eligibility were more likely to complete Wave 4 than were enrollees who were originally recruited through building or employer lists. Since symptoms and conditions were consistently more common among self-identified than list-identified enrollees in the current study, our results may tend to overestimate the prevalence of these conditions. On the other hand, because participants with PTSD at enrollment were less likely to remain active in the Registry than enrollees without PTSD, we may have underestimated the prevalence of PTSD among those affected. However, 9/11-related exposures were similar among Wave 4 participants and non-participants, suggesting that our findings on the relationships between such exposures and health end-points do not suffer from systematic bias due to differential attrition of this nature.

The current study addresses only the most common conditions that are associated with 9/11 exposure; we did not include other 9/11-related conditions, such as sarcoidosis, that are less common, yet potentially severe or even fatal, or various types of cancer which are being investigated as potentially associated with 9/11 exposure. Our description of comorbidities does not represent the complexity of the interrelationships among these conditions, an area of study which will be developed further in subsequent analyses. The full spectrum of the down-stream impacts of 9/11-related conditions, such as early retirement [23] or other measures of lost productivity, is also not examined here.

The persistently high prevalence of asthma, GERD, PTSD and depression 15 years after 9/11 and the association of these conditions with poor HRQOL provide strong support for the continuation of both medical care and health monitoring for 9/11-exposed persons. The poorer HRQOL outcomes and high levels of unmet mental health care need among those with mental health conditions in this population, despite the availability of care for 9/11-related mental health conditions through the WTC Health Program, are concerning. Outreach to inform exposed persons of available health resources, focusing on sub-groups with a particularly high prevalence of mental health conditions, is essential. Long-term monitoring of this cohort can inform preparation for and response to other complex disasters.

## Appendix

**Table 5:** Comparison of participants and non-participants in Wave 4

	Participants		Non-Participants		
Characteristic	N	%	N	%	*p*-value
Overall	36,864	54.6	30,640	45.4	
Sex					
Male	22,291	60.5	18,280	59.7	.03
Female	14,573	39.5	12,360	40.3	
Age on 9/11					
<18	886	2.4	1461	4.8	<.01
18–24	1776	4.8	2561	8.4	
25–44	18,840	51.1	17,527	57.4	
45–64	14,453	39.2	8082	26.5	
65+	891	2.4	912	3.0	
Race/ethnicity					
White	25,682	69.7	16,896	55.1	<.01
Black or African American	3554	9.6	4539	14.8	
Hispanic or Latino	4204	11.4	4922	16.1	
Asian	2134	5.8	2739	8.9	
Other	1290	3.5	1544	5.0	
Education					
Less than high school	8200	22.4	9191	30.7	<.01
At least some college	8999	24.6	7148	23.9	
College or higher	19,384	53.0	13,617	45.5	
Income					
Less than $25,000	2932	8.8	3938	14.8	<.01
$25,000 - $74,999	13,644	41.1	11,725	44.2	
$75,000 - $150,000	12,483	37.6	7892	29.8	
$150,000 or more	4169	12.6	2976	11.2	
Smoking status					
Never	21,019	57.8	17,566	59.3	<.01
Former	10,311	28.3	6774	22.9	
Current	5060	13.9	5267	17.8	
Self identified					
Yes	27,684	75.1	19,840	64.8	<.01
No	9180	24.9	10,800	35.3	
Registry eligibility group					
Rescue/recovery worker	16,976	46.7	12,602	41.8	<.01
Lower Manhattan area resident	5361	14.8	6237	20.7	
Area workers and school staff/students	12,600	34.7	10,000	33.2	
Passersby	1417	3.9	1310	4.4	
Dust cloud exposure					
Yes	19,121	52.1	15,602	51.2	.02
No	17,575	47.9	14,873	48.8	
Personally witnessed trauma on 9/11					
Yes	25,130	69.5	20,849	69.7	.63
No	11,041	30.5	9085	30.4	
Asthma at Wave 1					
Yes	5191	14.1	4266	14.0	.69
No	31,586	85.9	26,193	86.0	
GERS ^a^ at Wave 1					
Yes	11,202	30.5	9237	30.4	.67
No	25,484	69.5	21,166	69.6	
PTSD ^b^ at Wave 1					
Yes	5386	15.0	5285	18.2	<.01
No	30,629	85.1	23,690	81.8	

^a^Gastroesophageal reflux symptoms (GERS) were defined as reported heartburn, indigestion, or reflux at the time of the Wave 1 questionnaire

^b^Posttraumatic stress disorder (PTSD) was defined as a score > 44 on the 17-item, event-specific PTSD Checklist

## Abbreviations

9/11: September 11, 2001 World Trade Center terrorist attacks
GERD: gastroesophageal reflux disease
GERS: gastroesophageal reflux symptoms
HRQOL: health-related quality of life
PTSD: posttraumatic stress disorder
WTC: World Trade Center
